# Anemia-Driven Phenotypes in Lung Cancer: Linking Inflammation and Sarcopenia

**DOI:** 10.3390/diagnostics16111600

**Published:** 2026-05-23

**Authors:** Claudia Raluca Mariean, Oana Mirela Tiucă, Cristina Flavia Al-Akel, Sofia Teodora Muntean, Diana Maria Chiorean, Ovidiu Simion Cotoi

**Affiliations:** 1 Pathophysiology Department, George Emil Palade University of Medicine, Pharmacy, Science, and Technology of Targu Mures, 540142 Targu Mures, Romania; 2 Department of Radiology, Targu Mures County Emergency Hospital, 540142 Targu Mures, Romania; 3 Dermatology Department, George Emil Palade University of Medicine, Pharmacy, Science, and Technology of Targu Mures, 540142 Targu Mures, Romania; 4 Dermatology Clinic, Mures Clinical County Hospital, 540342 Targu Mures, Romania; 5 Department of Cardiovascular Surgery, Emergency Institute for Cardiovascular Diseases and Transplantation of Târgu Mures, 540136 Targu Mures, Romania; 6 Pathology Department, Mures Clinical County Hospital, 540011 Targu Mures, Romania

**Keywords:** anemia, lung cancer, inflammation, sarcopenia, phenotypes

## Abstract

**Background/Objectives**: Lung cancer remains a leading cause of cancer-related mortality and is characterized by complex tumor–host interactions, including systemic inflammation, metabolic dysregulation, and immune imbalance. This study aimed to evaluate whether a diagnosis of anemia reflects underlying inflammatory burden and to explore phenotype-based interactions between anemia, inflammation, and muscle depletion in lung cancer patients. **Methods**: A retrospective cohort study was conducted, including 70 patients diagnosed with lung cancer between 2019 and 2023. Anemia was defined using standard hemoglobin thresholds (<12 g/dL in women, <13 g/dL in men). Systemic inflammation was assessed using complete blood count-derived indices (NLR, PLR, SII, SIRI, and AISI), both individually and combined into a cumulative inflammatory score. Sarcopenia was evaluated through CT-based quantification of skeletal muscle area at the L3 level. Patients were stratified into four phenotypes based on anemia status, inflammatory burden, and sarcopenia. Statistical analyses like Mann–Whitney U, Kruskal–Wallis with Dunn post hoc testing, and univariate logistic regression were used. **Results**: Anemia was present in 44.3% of patients and was associated with a significantly higher inflammatory score compared to non-anemic patients (median 5 [IQR 4–5] vs. 4 [3–5], *p* = 0.024). Among inflammatory markers, PLR was significantly associated with anemia (OR = 4.94, 95% CI: 1.57–15.52, *p* = 0.004). The cumulative inflammatory score showed a non-significant association with anemia (OR = 1.28, 95% CI: 0.93–1.75, *p* = 0.124). Phenotype-based analysis revealed significant differences in skeletal muscle area (*p* = 0.004), with the sarcopenic-inflammatory phenotype exhibiting significantly lower muscle mass compared to other groups. No associations were observed between phenotypes and tumor stage or histological subtype. **Conclusions**: Anemia in lung cancer patients is closely associated with systemic inflammation and may reflect underlying biological vulnerability rather than tumor-specific characteristics. A phenotype-based approach integrating anemia, inflammatory markers, and sarcopenia provides a more comprehensive understanding of disease heterogeneity and may improve risk stratification. Further studies are needed to validate these findings and assess their prognostic implications.

## 1. Introduction

Lung cancer remains one of the leading causes of cancer-related mortality worldwide, accounting for a substantial proportion of cancer deaths despite advances in diagnosis and treatment. It is often diagnosed in advanced stages, when treatment options are limited, and systemic manifestations of the disease are already present [1]. Bronchopulmonary cancer involves complex pathophysiological mechanisms, leading to profound interactions between the tumor and the host, including chronic inflammation, metabolic alterations, and immune dysregulation. These systemic processes contribute to disease progression and the development of clinical features such as sarcopenia and cachexia [2,3].

Anemia is a frequent comorbidity in cancer patients, both at diagnosis and during disease progression, and has been consistently associated with adverse clinical outcomes [4]. Its prevalence ranges between 30 and 40% at diagnosis, increasing substantially in advanced disease or during treatment [5]. This variability is largely driven by underlying systemic inflammatory processes related to tumor–host interactions. Chronic systemic inflammation plays a central role in the development of cancer-related anemia through cytokine-mediated pathways, particularly involving the interleukin-6 (IL-6)–hepcidin axis. IL-6 stimulates hepatic hepcidin synthesis, which promotes the internalization and degradation of ferroportin, the major cellular iron exporter. Consequently, intestinal iron absorption and iron release from macrophages are reduced, leading to iron sequestration and functional iron deficiency despite preserved iron stores [6,7]. In addition, inflammatory cytokines may directly impair erythropoiesis and reduce erythropoietin responsiveness, further contributing to the development of cancer-related anemia [6,8].

Inflammatory indices derived from complete blood count (CBC) parameters, such as the neutrophil-to-lymphocyte ratio (NLR) and platelet-to-lymphocyte ratio (PLR), are widely used as markers of systemic inflammation [9]. The systemic immune–inflammation index (SII), which integrates neutrophils, platelets, and lymphocytes, has also been proposed as a marker of systemic inflammatory status and has been associated with disease severity and prognosis in cancer patients. These inflammatory markers have been associated with tumor progression, treatment response, and overall survival in multiple malignancies, including lung cancer [10]. Additionally, inflammatory pathways are linked to muscle catabolism, thus promoting the development of sarcopenia.

Sarcopenia plays an important role in the evaluation of oncologic patients, as it reflects the progressive loss of skeletal muscle mass and function [11]. It is considered a central component of cancer cachexia, a multifactorial syndrome characterized by muscle wasting, systemic inflammation, and metabolic alterations [12], and has been associated with decreased treatment tolerance and reduced survival [12,13,14]. These findings highlight the clinical relevance of muscle depletion as a marker of systemic disease and its potential impact on patient outcomes. Proinflammatory cytokines, including IL-6 and TNF-α, contribute to muscle catabolism by activating proteolytic pathways and promoting cachexia-related mechanisms, leading to progressive skeletal muscle depletion [14]. Consequently, systemic inflammation may represent a biological link between anemia and sarcopenia, both of which may reflect parallel manifestations of underlying metabolic and inflammatory dysregulation in cancer patients.

However, the relationship between anemia and sarcopenia remains incompletely understood, with inconsistent evidence regarding whether these conditions are directly linked or arise independently from systemic inflammation. In addition, most studies have evaluated these parameters individually rather than in an integrated manner, limiting the understanding of their combined biological impact [15,16,17]. This highlights the need for integrative approaches that combine hematologic, inflammatory, and metabolic parameters to better characterize disease biology.

Phenotype-based approaches integrating anemia, inflammatory burden, and sarcopenic status may provide a more comprehensive understanding of patient heterogeneity and disease severity by identifying biologically distinct subgroups. Although the associations between anemia, systemic inflammation, sarcopenia, and cancer have been described individually, previous studies have mainly focused on these factors as prognostic markers or clinical variables rather than within an integrated biological framework [7,18]. Consequently, the combined impact of hematologic, inflammatory, and metabolic alterations on systemic vulnerability in lung cancer patients remains insufficiently characterized. In recent years, increasing attention has been directed toward multidimensional approaches integrating systemic inflammation, body composition, and host-related factors to better reflect disease heterogeneity and patient outcomes [19].

In this context, the primary objective of this study was to investigate whether anemia could serve as a marker of underlying systemic inflammation in patients with lung cancer. In addition, we aimed to develop a phenotype-based classification integrating anemia status, inflammatory burden, and sarcopenic changes into predefined biologically relevant categories. To our knowledge, few previous studies have explored these parameters simultaneously within an integrated phenotype-based framework in lung cancer patients. These anemia-driven phenotypes were constructed a priori based on clinically meaningful hematologic, inflammatory, and metabolic characteristics of patients to identify subgroups with varying degrees of systemic involvement and biological severity. Such an approach may facilitate the early identification of patients at increased risk of an unfavorable biological course and support more individualized multidisciplinary management strategies, supportive interventions, and closer clinical monitoring.

## 2. Materials and Methods

The present study was designed as a retrospective, cross-sectional observational study including 70 patients diagnosed with lung carcinoma between 1 January 2019 and 31 December 2023, at the Clinical County Hospital Mureș, Târgu Mureș, Romania. Given the study’s retrospective and exploratory nature, no formal sample size or power calculation was performed. The analysis included all consecutive eligible patients meeting the predefined inclusion criteria during the study period. Eligible participants were adults (>18 years) with histopathologically confirmed lung carcinoma, available laboratory parameters required for analysis, and a native CT examination including the L3 vertebral level. Patients presenting active infections, concomitant malignancies, known hematological disorders at the time of diagnosis, lack of histopathological confirmation, incomplete laboratory data, or absence of a CT examination, including the L3 vertebral level, were excluded from the study.

The study was conducted in accordance with the principles of the Declaration of Helsinki and was approved by the Ethics Committee of the Clinical County Hospital Mureș (approval no. 20419/15 December 2023).

### 2.1. Definition of the Analyzed Parameters

Clinical, biological, and imaging data were collected from institutional records.

❖ Anemia was defined based on the following clinical thresholds: a hemoglobin (Hb) level < 12 g/dL in women and <13 g/dL in men [7].
❖ The inflammatory status at the time of initial diagnosis was assessed based on the analysis of parameters derived from their CBCs. The following CBC-derived inflammatory indexes were calculated: the neutrophil-to-lymphocyte ratio (NLR), platelet-to-lymphocyte ratio (PLR), systemic inflammatory index (SII), systemic inflammatory response index (SIRI), and aggregate index of systemic inflammation (AISI). Each marker was dichotomized (0/1) based on predefined cut-off values derived from the literature. The formulas used for these parameters are presented in Table 1.

❖ A cumulative inflammatory score (range 0–5) was proposed and calculated by summing the number of elevated inflammatory indices, assigning one point for each marker above its cut-off value. Each inflammatory index was dichotomized based on previously reported cut-off values. The selected cut-off values were based on previously published studies evaluating systemic inflammatory markers in oncologic populations, particularly in lung cancer patients, and were chosen to ensure consistency with existing literature and clinical interpretability. Values above the cut-off were coded as 1 (elevated), and those within or below the normal range as 0. Based on preexisting literature data, the cut-off values were defined as follows: NLR: 0.78–3.53; PLR: <185; SII: 660 (NSCLC)/1600 (SCLC); AISI: 351; SIRI: 2;
❖ Sarcopenia was assessed on baseline CT examinations performed at the time of diagnosis by measuring the skeletal muscle cross-sectional area (SMA) at the level of the third lumbar vertebra (L3). Image analysis was performed using the ODIASP software tool (version 2.2.9), which automatically identified and quantified skeletal muscle area on axial CT images [20,21,22,23]. The analyzed muscle compartment at the L3 level included the psoas, paraspinal muscles, transversus abdominis, rectus abdominis, and the internal and external oblique muscles. In accordance with previously validated CT-based body composition assessment methods. Patients were classified as sarcopenic according to previously reported sex-specific SMA cut-off values (<92.2 cm^2^ for females and <144.3 cm^2^ for males) [24].
❖ The tumor stage at diagnosis: the patient’s tumor stage was established, considering the 8th edition of the TNM grading system of malignant tumors.
❖ The histological type of lung carcinoma: NSCLC (adenocarcinoma, squamous cell carcinoma, adenosquamous carcinoma, and NSCLC that is not otherwise specified (NOS)) and SCLC.
❖ The smoking status was assessed based on the anamnesis.

### 2.2. Biological Phenotype and Associated Classification

Based on anemia status, associated inflammatory response, and sarcopenic changes, patients were stratified into four biologically defined phenotypes:

❖ Non-anemic and low-inflammatory profile: normal Hb and inflammatory score ≤ 1.
❖ Inflammation-driven anemia: low Hb and cumulative inflammatory score ≥ 3, without associated sarcopenia.
❖ Sarcopenic inflammatory anemia: low Hb, cumulative inflammatory score ≥ 3, and associated sarcopenia.
❖ Intermediate profile: patients not meeting the criteria for the above categories.

### 2.3. Statistical Analysis

Comparisons between two independent groups (anemic vs. non-anemic) were performed using the Mann–Whitney U test. Comparisons across multiple phenotypes were performed using the Kruskal–Wallis test, with post hoc pairwise comparisons conducted using Dunn’s test with Bonferroni correction.

Associations between categorical variables were assessed using the chi-square test or Fisher’s exact test, depending on expected cell counts.

Continuous variables were expressed as medians and interquartile ranges (IQRs) due to non-normal distributions.

Univariate logistic regression analysis was performed to evaluate the association between systemic inflammation and anemia, with results expressed as odds ratios (ORs) and 95% confidence intervals (CIs). Two models were constructed for the same outcome (anemia): a cumulative inflammatory model using the cumulative inflammatory score as a continuous predictor and individual marker models, in which each inflammatory index (NLR, PLR, SII, SIRI, and AISI) was entered separately as a binary predictor. All statistical tests were two-sided, and *p*-values < 0.05 were considered statistically significant. Given the relatively small sample size and the exploratory, retrospective design of the study, multivariable adjustment was not performed to minimize the risk of model overfitting and unstable statistical estimates. Therefore, the statistical analysis focused on univariate exploratory associations to identify potential biological relationships among anemia, systemic inflammation, and sarcopenia. In addition, given the exploratory nature of the study and the number of comparisons, results were interpreted in the context of overall biological consistency rather than isolated statistical significance.

## 3. Results

### 3.1. Characterization of the Study Population

❖ General Characteristics

**Figure 1:**
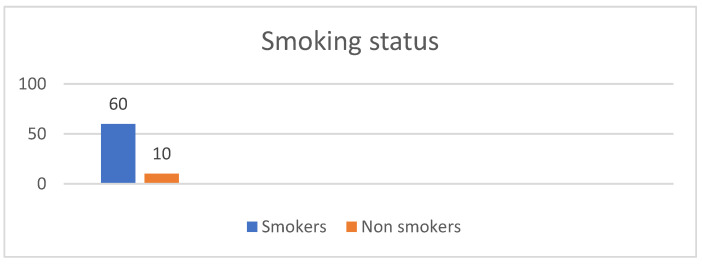
Smoking status of the study population.

In the current study, 70 patients diagnosed with lung carcinoma were included. The majority were males (53). The median age at diagnosis was 67.3 years. Most patients lived in rural areas (37 patients). Regarding the smoking status, our results showed that the vast majority of lung cancer patients were smokers (60 patients), as presented in Figure 1.

❖ The Histological Type of the Tumor

**Figure 2:**
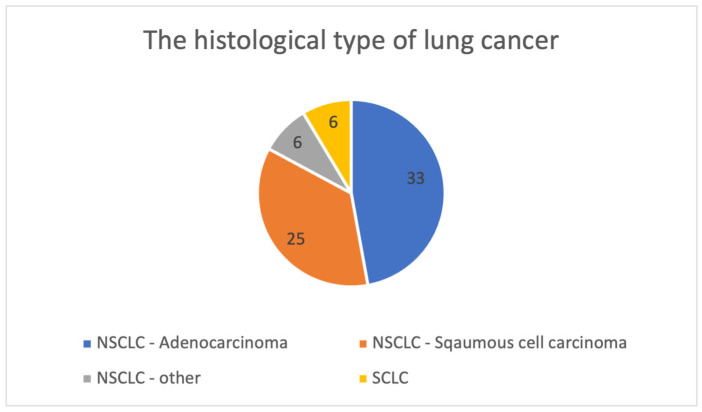
Histopathological classification of lung cancer.

Of the 70 patients in the study population, 6 had SCLC and 64 had NSCLC. Adenocarcinoma was the most diagnosed histological type of NSCLC (33 patients). Squamous cell carcinoma was found in 25 patients, while 6 patients were diagnosed with other types of NSCLC (adenosquamous and NOS carcinoma). The distribution of histological subtypes is shown in Figure 2.

❖ The TNM Stage at Diagnosis

**Figure 3:**
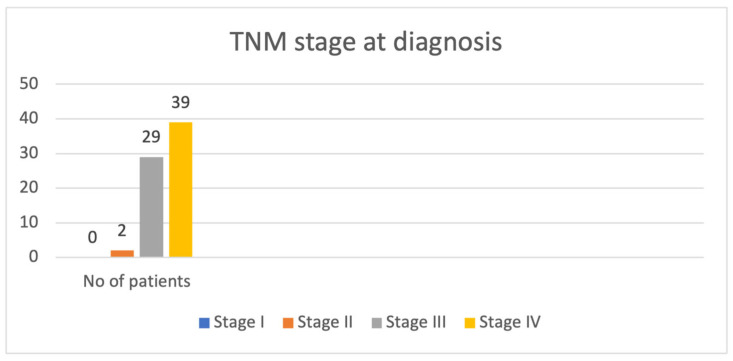
TNM stage of the tumors.

Figure 3 presents the stage at diagnosis among lung cancer patients: stage IV, 39 patients; stage III, 29 patients; stage II, 2 patients; and no patients in stage I.

### 3.2. Anemia and Its Association with Cumulative Inflammatory Score and Individual Inflammatory Indices

**Figure 4:**
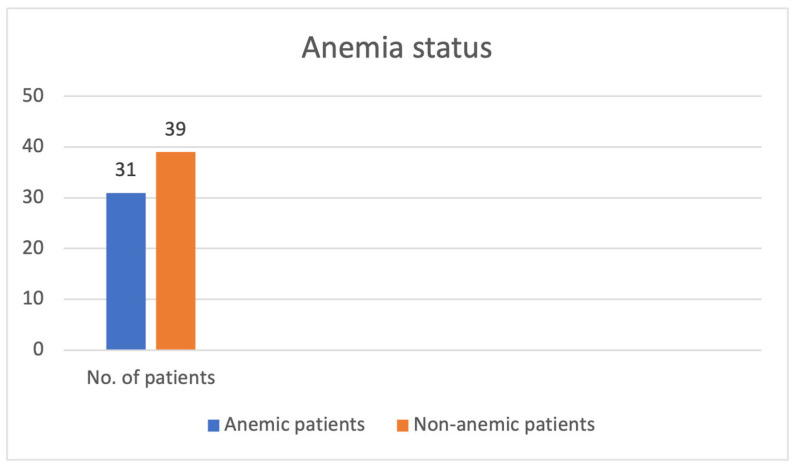
Anemia status in the study population.

Patients were divided into two groups based on hemoglobin status (anemic vs. non-anemic). 31 patients (44.3%) were classified as anemic and 39 (55.7%) as non-anemic, as presented in Figure 4.

The relationship between anemia and the inflammatory profile of the study population was assessed using both the cumulative inflammatory score and each inflammatory marker (NLR, PLR, SII, SIRI, and AISI) separately.

The cumulative inflammatory score was significantly higher in anemic patients compared to non-anemic patients (median 5 [IQR 4–5] vs. 4 [3–5], *p* = 0.024), indicating an increased inflammatory burden in the anemic group. It was highly prevalent within phenotypic subgroups characterized by elevated inflammatory burden, particularly in patients with inflammatory scores ≥ 3. Among individual inflammatory markers, PLR was significantly associated with anemia, with approximately fivefold increased odds. In contrast, NLR, SII, SIRI, and AISI did not reach statistical significance. These results are summarized in Table 2.

To further explore this relationship, univariate logistic regression analysis was performed using anemia as the dependent variable and the cumulative inflammatory score as a continuous predictor. Although the association did not reach statistical significance (OR = 1.28 per unit increase, *p* = 0.124, CI: 0.93–1.75), a non-significant trend was observed, suggesting that a higher inflammatory burden may be associated with an increased likelihood of anemia.

No significant association was observed between anemia and histopathological subtype (*p* = 0.77), suggesting that anemia is not specific to a particular tumor type. In addition, anemia tended to be more frequent in advanced tumor stages, particularly stage IV, although this did not reach statistical significance (*p* = 0.125).

### 3.3. Sarcopenia Assessment in the Study Population

**Figure 5:**
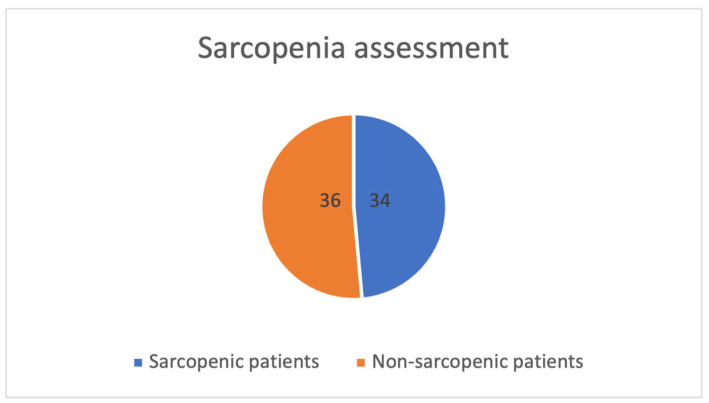
Sarcopenia status in the study population.

The skeletal muscle area at the level of the third lumbar vertebra (L3SMA) was measured at the time of initial diagnosis using the ODIASP software to classify patients as sarcopenic/non-sarcopenic based on these findings. Our results showed that the number of sarcopenic patients was almost the same as the number of non-sarcopenic ones: 34 patients were sarcopenic at the time of lung cancer diagnosis, while 36 were not, as shown in Figure 5.

### 3.4. Phenotype-Based Analysis

The last step of the study was to stratify the patients into biologically defined phenotypes based on anemia, inflammatory burden, and sarcopenia.

Table 3 presents the main characteristics of the phenotypes included in the analysis.

When patients were stratified into four biologically defined phenotypes, significant differences were observed in both inflammatory response and skeletal muscle area. Table 4 summarizes the mean values of both the inflammatory score and skeletal muscle area for the four phenotypes we have proposed.

The cumulative inflammatory score varied across phenotypes, as expected based on the predefined classification criteria, with the lowest values observed in the low-inflammatory non-anemic group and the highest in the inflammatory phenotypes. The intermediate group showed heterogeneous values.

Skeletal muscle area differed significantly between phenotypes (*p* = 0.004). Post hoc pairwise comparisons revealed that patients with sarcopenic-inflammatory anemia had significantly lower SMA values compared to both the inflammatory anemia group (*p* = 0.029) and the intermediate group (*p* = 0.011). No significant differences were observed between the other phenotype groups. Although reduced skeletal muscle area is inherent to the definition of sarcopenia, these findings highlight that patients with sarcopenic-inflammatory anemia represent a distinct biological subgroup characterized by more pronounced muscle depletion. Notably, patients with inflammatory anemia without sarcopenia exhibited relatively preserved muscle mass despite a high inflammatory burden, suggesting that inflammation alone does not fully account for muscle loss.

In contrast, no significant differences were observed in tumor stage (*p* = 0.65) or age (*p* = 0.27) between the four phenotypes.

## 4. Discussion

Anemia was observed in 44.3% of patients at the time of initial diagnosis, a prevalence consistent with previously reported data in lung cancer populations [25]. Studies have shown that among patients with solid tumors, those diagnosed with lung cancer may present one of the highest risks of developing anemia during the course of the disease, with reported prevalence rates reaching up to 50–70% [26].

Inflammation is known to be closely associated with the development of anemia in cancer patients. Our findings are consistent with these observations, as anemic patients in the present study exhibited significantly higher cumulative inflammatory scores compared to non-anemic patients, supporting the relationship between anemia and systemic inflammation in lung cancer patients [27,28,29]. Proinflammatory cytokines such as interleukin-6 stimulate hepcidin production, the key regulator of iron homeostasis, leading to inhibition of intestinal iron absorption and sequestration of iron within macrophages, thereby promoting functional iron deficiency and impaired erythropoiesis [8,28,30]. In addition, systemic inflammation alters iron metabolism, erythropoiesis, and red blood cell survival through multiple interconnected pathways [26]. Inflammatory mediators may also suppress erythropoiesis by inhibiting erythroid progenitor proliferation and reducing responsiveness to erythropoietin stimulation, further contributing to decreased hemoglobin levels [8]. Furthermore, oxidative stress and inflammatory mediators may affect red blood cell membrane dynamics, rigidity, and permeability, potentially impairing erythrocyte function. The interaction between red blood cells and reactive oxygen species (ROS) appears particularly relevant, as excessive ROS production has been associated with mechanical alterations of erythrocytes [31,32,33]. Therefore, anemia in the oncologic setting may reflect systemic immune activation and inflammatory dysregulation rather than an isolated hematologic abnormality.

In addition, systemic inflammation may influence both the quantity and quality of skeletal muscle mass [34]. Proinflammatory cytokines promote proteolysis and inhibit protein synthesis in skeletal muscle, contributing to sarcopenia while simultaneously impairing erythropoiesis, suggesting that systemic inflammation may represent a common biological link between both processes [35,36]. In this context, anemia should not be interpreted exclusively as an isolated hematologic abnormality, but rather as a potential marker of chronic inflammatory activation and systemic disease burden.

Platelets are increasingly recognized as active participants in tumor-associated inflammation, contributing to cytokine release, immune modulation, tumor progression, and metastasis [37,38]. In this context, platelet-related indices, such as the platelet-to-lymphocyte ratio (PLR), have emerged as potentially relevant biomarkers of systemic inflammation and have shown a significant association with anemia in the present cohort, suggesting a possible role for platelet-related inflammatory pathways in cancer-associated anemia. PLR integrates both inflammatory activation and immune dysregulation into a single composite marker [4,39,40,41]. The association observed between PLR and anemia in our study may reflect the interplay between platelet activation, cytokine production, and iron metabolism.

In our study, PLR demonstrated a stronger association with anemia than other inflammatory indices, suggesting that platelet-related inflammatory mechanisms could play a potentially relevant role in cancer-related systemic dysregulation. According to Li et al., platelets play an important role in cancer progression by modulating immune responses, enhancing antigen presentation, and promoting interactions between immune cells. In addition, tumor-activated platelets contribute to angiogenesis, increase vascular permeability, and facilitate tumor cell migration. They may also promote thrombosis through interactions with red blood cells, thereby potentially contributing to adverse outcomes in cancer patients [42]. In contrast, other inflammatory markers, such as NLR, SII, SIRI, and AISI, did not show statistically significant associations when analyzed individually. These findings may support the potential utility of composite or pathway-specific biomarkers when evaluating systemic inflammation in oncology patients.

Furthermore, although anemia did not reach statistical significance as an independent predictor of biological severity, it demonstrated a consistent trend toward increased severity, suggesting a potentially clinically relevant association. The lack of statistical significance may be due to the relatively small sample size; however, the observed trend suggests that anemia could still have clinically relevant implications when interpreted in the appropriate biological context. No significant association was identified between anemia and tumor characteristics, such as histopathological subtype or tumor stage, suggesting that the observed hematological alterations may be more closely related to host systemic changes than to tumor-specific characteristics. These observations are consistent with previous studies that did not identify a direct association between anemia and the histopathological subtype of lung cancer [43,44,45].

When assessing sarcopenic changes, sarcopenia tended to be more frequent among patients with elevated inflammatory burden, suggesting a potential relationship between inflammation and muscle depletion. These findings may support the hypothesis that anemia and sarcopenia could represent parallel manifestations of systemic inflammation rather than strictly sequential events [46]. Beyond simple muscle depletion, sarcopenia is increasingly recognized as a marker of systemic metabolic and inflammatory dysregulation in cancer patients. Previous studies demonstrated that sarcopenia is associated with reduced functional reserve, impaired tolerance to oncologic treatment, increased treatment-related toxicity, and poorer clinical outcomes [12,13]. Inflammatory cytokines such as IL-6 and TNF-α may contribute not only to skeletal muscle proteolysis but also to alterations in mitochondrial metabolism, energy balance, and nutritional status, thereby promoting the development of cancer cachexia [references]. In this context, the association observed between elevated inflammatory burden and sarcopenic changes in our cohort may support the concept that muscle depletion represents part of a broader host-related systemic response rather than an isolated nutritional alteration [12,13,47].

When extending the analysis to phenotype-based categories, important differences in biological response patterns were observed. As expected, based on the predefined classification criteria, the sarcopenic-inflammatory phenotype was characterized by both elevated inflammatory burden and reduced muscle mass, consistent with a cachexia-like profile that has previously been associated with poorer oncologic outcomes [48].

In contrast, the inflammatory anemia phenotype was characterized by elevated inflammatory burden but relatively preserved muscle mass, potentially suggesting that anemia may become detectable in association with systemic inflammation before overt sarcopenic changes are clinically evident. The intermediate phenotype demonstrated heterogeneous biological characteristics, possibly reflecting transitional or overlapping systemic alterations rather than a clearly distinct biological profile. Importantly, these phenotypes were not associated with tumor stage, suggesting that systemic biological alterations may be more closely linked to host-related inflammatory and metabolic responses than to local tumor characteristics alone [47].

Taken together, our findings suggest that anemia may be better understood within a broader biological framework integrating inflammation and metabolic status, rather than as an isolated clinical parameter.

In the context of precision oncology, increasing attention has recently been directed toward quantitative imaging approaches and radiomics as non-invasive tools for prognostic stratification and clinical decision support in lung cancer patients. Future integrative approaches combining inflammatory biomarkers, phenotype-based classifications, and advanced imaging analysis may further improve personalized prognostic evaluation in lung cancer patients [49,50].

This study has several limitations, including a relatively small sample size, a single-center design, and a retrospective analysis, all of which may limit the generalizability of the findings and reduce the statistical power to detect independent associations. In addition, follow-up data on patient outcomes were unavailable. Future prospective studies, including longitudinal follow-up and larger patient cohorts, are therefore warranted to further validate these observations. Despite these limitations, the present study provides an integrative exploratory analysis of anemia, inflammatory burden, and sarcopenia in lung cancer patients, offering additional insights into the complex interactions between systemic inflammation, metabolic alterations, and hematologic dysfunction in this population. Further prospective multicenter studies including longitudinal outcome data are required to validate the prognostic relevance and clinical applicability of the proposed phenotype-based framework.

## 5. Conclusions

Anemia in patients with bronchopulmonary cancer is closely associated with systemic inflammation and reflects underlying biological vulnerability rather than tumor-specific characteristics. While not an independent predictor of biological severity, anemia might serve as a screening tool to identify patients with an increased inflammatory response and to help define biologically distinct phenotypes, especially when combined with sarcopenic changes.

The integration of anemia, inflammatory indices, and muscle status provides a more comprehensive understanding of disease biology and may improve risk stratification in lung cancer patients, facilitating early identification of high-risk patients and enabling a more personalized therapeutic approach. Future studies with larger cohorts are needed to validate these findings and to explore the prognostic and therapeutic implications of phenotype-based approaches.

## Figures and Tables

**Table 1:** Formula of the inflammatory indices.

Parameter	Formula
Neutrophil-to-lymphocyte ratio (NLR)	Neutrophil count/lymphocyte count [×10^3^/µL] [18]
Platelet-to-lymphocyte ratio (PLR)	Platelet count/lymphocyte count [×10^3^/µL] [18]
Systemic Inflammatory Index (SII)	(Neutrophil count × platelet count)/lymphocyte count [×10^3^/µL] [19]
Systemic Inflammatory Response Index (SIRI)	(Neutrophil count × monocyte count)/lymphocyte count [×10^3^/µL] [20]
Aggregate index of systemic inflammation (AISI)	(Neutrophil count × monocyte count × platelet count)/lymphocyte count [×10^3^/µL] [20]

**Table 2:** Inflammatory status in the study population, as indicated by anemia.

Variable	Anemic Patients (*n* = 31)	Non-Anemic Patients (*n* = 39)	*p*-Value
Cumulative inflammatory score	5 (4–5)	4 (3–5)	0.024
NLR	5.76 (3.70–7.91)	4.00 (3.11–6.08)	0.108
PLR	317.24 (215.13–408.03)	194.15 (118.80–318.07)	0.004
SII	2061.93 (1387.26–3315.40)	1424.24 (965.77–2258.42)	0.88
SIRI	3.15 (2.41–4.73)	2.41 (1.94–3.79)	0.39
AISI	1155.28 (818.75–1840.95)	840.30 (537.12–1329.11)	0.48

**Table 3:** Main characteristics of the included phenotypes.

Phenotype	Definition	Inflammatory Burden	Muscle Mass (SMA)	Clinical Interpretation
1. Low-inflammatory non-anemic	Hb normal + inflammatory score ≤ 1	Low	Preserved	Baseline/low systemic involvement
2. Inflammatory anemia	Hb low + inflammatory score ≥ 3	High	Relatively preserved	Inflammation-driven anemia
3. Inflammatory–sarcopenic anemia	Hb low + inflammatory score ≥ 3 + sarcopenia	Very high	Reduced	Systemic involvement
4. Intermediate group	Other combinations	Intermediate/variable	Variable	Heterogeneous state

**Table 4:** Phenotype-based analysis of the median values of the inflammatory score and skeletal muscle area in the study population.

Phenotype	*n*	Inflammatory Score	Skeletal Muscle Area (SMA)
Low-inflammatory non-anemic	6	0 (0–0.75)	142 (116–153)
Inflammatory anemia	8	5 (4–5)	136.04 (109.64–151.22)
Sarcopenic-inflammatory anemia	19	5 (5–5)	103 (95.24–129.5)
Intermediate group	37	4 (3–5)	132 (114–152.37)

## Data Availability

No new data were created.

## Abbreviations

The following abbreviations are used in this manuscript:

CBC	Complete blood count
NLR	Neutrophil-to-lymphocyte ratio
PLR	Platelet-to-lymphocyte ratio
SII	Systemic Inflammatory Index
AISI	Aggregate index of systemic inflammation
SIRI	Systemic Inflammatory Response Index
CT	Computer tomography
L3	3rd lumbar vertebra
SMA	Skeletal muscle area
NSCLC	Non-small cell lung carcinoma
SCLC	Small-cell lung carcinoma
TNM	Tumor, node, metastasis
Hb	Hemoglobin
NOS	Not-otherwise-specified carcinoma
IQR	Interquartile range
OR	Odds ratio
CI	Confidence interval
RBCs	Red blood cells
